# A Combination of Nifedipine and OctreotideTreatment in an HyperinsulinemicHypoglycemic Infant

**DOI:** 10.4274/jcrpe.1230

**Published:** 2014-06-05

**Authors:** Erdem Durmaz, Sarah E. Flanagan, Mesut Parlak, Sian Ellard, Sema Akcurin, İffet Bircan

**Affiliations:** 1 Mersin State Hospital, Department of Pediatric Endocrinology, Mersin, Turkey; 2 Institute of Biomedical and Clinical Science, University of Exeter Medical School, Exeter, UK; 3 Antalya Training and Research Hospital, Department of Pediatric Endocrinology Clinic, Antalya, Turkey; 4 Akdeniz University School of Medicine, Department of Pediatrics, Pediatric Endocrinology Division, Antalya, Turkey

**Keywords:** Nifedipine, diazoxide, hypotension, hypoglycemia, Hyperinsulinism

## Abstract

Hyperinsulinemic hypoglycemia (HH) is the commonest cause of persistent hypoglycemia in the neonatal and infancy periods. Mutations in the ABCC8 and KCNJ11 genes, which encode subunits of the ATP-sensitive potassium channel in the pancreatic beta cell, are identified in approximately 50% of these patients. The first-line drug in the treatment of HH is diazoxide. Octreotide and glucagon can be used in patients who show no response to diazoxide. Nifedipine, a calcium-channel blocker, has been shown to be an effective treatment in a small number of patients with diazoxide-unresponsive HH. We report a HH patient with a homozygous ABCC8 mutation (p.W1339X) who underwent a near-total pancreatectomy at 2 months of age due to a lack of response to diazoxide and octreotide treatment. Severe hypoglycemic attacks continued following surgery, while the patient was being treated with octreotide. These attacks resolved when nifedipine was introduced. Whilst our patient responded well to nifedipine, the dosage could not be increased to 0.75 mg/kg/day due to development of hypotension, a reported side effect of this drug. Currently, our patient, now aged 4 years, is receiving a combination of nifedipine and octreotide treatment. He is under good control and shows no side effects. In conclusion, nifedipine treatment can be started in patients with HH who show a poor response to diazoxide and octreotide treatment.

## INTRODUCTION

Hyperinsulinemic hypoglycemia (HH) is the most common cause of recurrent or persistent hypoglycemia in infants (1). Its incidence is estimated to be 1 in 50 000 live births in outbred communities (2). Insufficient suppression of insulin secretion in the presence of recurrent serious hypoglycemia is the basic characteristic of this condition.

The most common mechanism underlying congenital hyperinsulinism is the dysfunction of the pancreatic ATP-sensitive potassium (K-ATP) channel. This channel consists of two subunits: the sulfonylurea receptor (SUR1) subunit and the inwardly rectifying K+ subunit (KIR 6.2) (3). Usually, closure of the K-ATP channel initiates the depolarization in beta cell membrane leading to opening of calcium channels, increase in intracellular calcium concentrations and dysregulation of insulin release (4). The most common known cause of HH is mutations (ABCC8 and KCNJ11) in the genes encoding the subunits of K-ATP channel. These mutations result in absence or dysfunction of the K-ATP channels on the cell surface, which causes unregulated insulin release (4,5). Diazoxide is the first-line treatment of choice for HH, although the clinical effectiveness of this drug is variable (6). Glucagon and calcium-channel blockers (nifedipine) can be considered in patients showing no response to diazoxide and octreotide. To date, a limited number of cases have been reported to have a good response to nifedipine. However, the underlying molecular pathophysiology of HH in these patients is not clear (5).

Here, we report a patient with a homozygous ABCC8 nonsense mutation who responded well to a combination of nifedipine and octreotide treatment following a near-total pancreatectomy.

## CASE REPORT

Our patient was a male infant with a birth weight of 5000 g, born at term by normal spontaneous vaginal delivery to a 31-year-old healthy mother. The parents were first-degree cousins. On postpartum day 2, hypoglycemia (24 mg/dL) and hyperinsulinism (insulin level 15 mU/L) were detected. The patient was treated with 15-20 mg/kg/min IV dextrose infusion and 15 mg/kg/day of diazoxide. Steroid treatment (2 mg/kg/day) was also administered for a total of three days. Subsequently, during hypoglycemic episodes, octreotide (25 µg/kg/day) was introduced while diazoxide dosage was tapered down. The blood glucose levels remained at low levels and the patient underwent a near-total pancreatectomy at the age of 2 months. 

Hypoglycemic episodes were noted postoperatively and octreotide treatment (15 µg/kg/day) was re-started. As the hypoglycemia persisted despite an increase in the octreotide dose to 30 µg/kg/day (divided into 3 doses), a short-acting nifedipine was added (0.25 mg/kg/day (divided into 3 doses) to the treatment. Under close blood pressure and electrocardiography monitoring, the dose of nifedipine was increased to 0.5 mg/kg/day (3 doses a day) and a good glycemic control was obtained, especially in postprandial blood glucose levels. The octreotide dose was tapered down to 5 µg/kg/day, but the treatment could not be discontinued. The dosage of nifedipine was subsequently increased to 0.75 mg/kg/day, but the dose was reverted to 0.5 mg/kg/day due to low systolic blood pressure. 

The patient has been receiving octreotide therapy (5 µg/kg/day) for 4 years and nifedipine treatment (0.5 mg/kg/day) for 3 years. At present, he is in a state of good glycemic control with no severe hypoglycemic episodes. Glucose monitoring is made by a home glucometer, with routine venous hemoglobin A1c, insulin and glucose level measurements every three months. The patient is now four years old, his growth status is within normal limits (weight and height are both at the 25th percentile) and no problem is noted in his neuromotor development. No drug side effects were observed and thyroid function tests remained within normal limits. Genetic investigation revealed a previously reported homozygous nonsense mutation (p.W1339X; c.4017G>A) in the ABCC8 gene (7). This result was consistent with the histological findings, which identified diffuse disease in the resected pancreas.

## DISCUSSION

Our patient responded well to nifedipine treatment. Nifedipine is a calcium-channel blocker and has been used in some patients with HH with success, although the vast majority of patients fail to show any response (8,9). The underlying molecular pathophysiology of HH is still unclear (5). Darendeliler et al (10) screened 13 HH patients for mutations in ABCC8 and KCNJ1. A genetic etiology could not be identified in 10 of these patients. Two out of three patients with ABCC8 mutations underwent pancreatectomy because of lack of response to treatment and hypoglycemia was reported to occur also post-operatively. Hyperinsulinism recurred after surgery in two patients. Treatment with either diazoxide or calcium-channel blocker proved to be effective in controlling hypoglycemia in the rest of these children. The authors concluded that mutations in the ABCC8 gene may not be predictive of the response to drugs. 

Another unclear point is nifedipine dose. Although some patients respond to a dose of 0.25 mg/kg/day, the dose need to be increased up to 2 mg/kg/day in others (8,9,11,12). This situation may be related to mutation type. In our case, there was no response to nifedipine in a dose of 0.25 mg/kg/day, whereas a dramatic response was obtained with 0.5 mg/kg/day. We could not increase the dosage due to hypotensive attacks which occurred at 0.75 mg/kg/day. Hypotension is a possible, yet uncommon side effect of nifedipine treatment in HH (5). Based on our experience with this patient, we suggest that the dose increase during nifedipine treatment for hyperinsulinism should be done with careful monitoring for undesirable effects.

Octreotide is a long-acting analogue of the natural hormone somatostatin, which has inhibitory effects on the release of insulin from pancreatic beta cells. It is used in the short- and long-term management of some patients with HH (13). Octreotide has been successfully used in the long-term management of some patients with HH in combination with frequent feeding (14). 

As far as we know, our patient is the first case to be treated with a combination of nifedipine and octreotide. Loechner et al (15) reported a patient diagnosed with congenital hyperinsulinism (due to KCNJ11 gene mutation), who had hypertension and was treated with octreotide and amlodipine (a calcium-channel blocker). They concluded that with this treatment, the baseline mean blood glucose level increased and was stabilized at a higher level than it would be by octreotide alone and that no episodes of blood glucose levels <60 mg/dL occurred with octreotide plus amlodipine (15). Voltage-gated T-type and L-type Ca2+ channels as well as Na+ channels participate in glucose-stimulated electrical activity and insulin secretion in pancreatic beta cells (16). Nifedipine is an L-type calcium-channel blocker. Octreotide inhibits Ca2+ entry into pancreatic beta cells via voltage-operated Ca2+ channels (VOCCs) of the L-type, leading to suppression of insulin secretion (17). Moreover, octreotide hyperpolarizes insulinoma cells by activating (K-ATP) channel and thus lowers insulin secretion (18). Although the mechanism is not clear, octreotide may have an additive effect on nifedipine treatment via effecting VOCCs and (K-ATP) channels. In our case, no side effect related to octreotide and nifedipine treatment was observed during the 3-year follow-up period.

In conclusion, our case of diazoxide-resistant hyperinsulinism due to homozygous ABCC8 nonsense mutation showed a good response to a combined treatment of nifedipine and octreotide. Further studies are required to evaluate the nifedipine effect and its dose range in patients with HH.

**Acknowledgements**

Sian Ellard is employed by the Exeter Clinical Research Facility and is a Wellcome Trust Senior Investigator. The genetic testing was funded by a research grant from the Medical Research Council.
